# Convergence between the dimensional PD models of ICD-11 and DSM-5: a meta-analytic approach

**DOI:** 10.3389/fpsyt.2023.1325583

**Published:** 2023-11-30

**Authors:** Luis Hualparuca-Olivera, Tomás Caycho-Rodríguez, Julio Torales, Dayana Ramos-Campos

**Affiliations:** ^1^Escuela de Psicología, Universidad Continental, Huancayo, Peru; ^2^Facultad de Psicología, Universidad Científica del Sur, Lima, Peru; ^3^Department of Medical Psychology, School of Medical Sciences, Universidad Nacional de Asunción, San Lorenzo, Paraguay; ^4^Department of Research, Psychological Science, Uncanny, Huancayo, Peru

**Keywords:** ICD-11, DSM-5, personality disorder, dimensional models, severity, traits, convergent validity, meta-analysis

## Abstract

In the current diagnostic systems, the International Classification of Diseases-11th rev. (ICD-11) and the Diagnostic and Statistical Manual of Mental Disorders-5th ed. (DSM-5), the evaluation and diagnosis of personality disorder (PD) aim at dimensional examination of the severity of its dysfunction and the stylistic features that accompany it. Since their implementation, or even before, several measures have been developed to assess PD severity and traits in both models. Thus, convergent validity metrics have been reported with various PD measures; however, the convergence of the same constructs included in the measures of these two models remains undefined. The objective of the present review was to examine whether there is a sufficient relationship between PD measures of the ICD-11 and DSM-5 AMPD in the general population. For this meta-analytic review, systematic searches were conducted in Web of Science, PubMed, Scopus, and Google Scholar. We included studies that reported Pearson’s *r* correlations without restrictions on language, age, sex, setting, type of sample, or informant of the measures. We excluded associations with anankastia, psychoticism or the borderline pattern because they were not comparable between one dimensional model and the other. We examined the quality of the evidence with the JBI Critical Appraisal Checklist for Analytical Cross Sectional Studies, and performed the random effects meta-analysis with the ‘meta’ package of the RStudio software. Of the 5,629 results returned by the search, 16 studies were eligible; and showed moderate quality. The risk of bias was manifested by not specifying the details of the sample, the recruitment environment, and the identification and control of confounding factors. Thirteen studies provided two or more correlations resulting in a total of 54 studies for meta-analysis. The overall effect size estimate (correlation) was moderate for the overall model (*r* = 0.62, 95% CI [0.57, 0.67], *p* < 0.0001, *I*^2^ = 97.6%). For the subgroup of associations, ICD-11 severity model and DSM-5 AMPD severity model, the correlation was also moderate (*k* = 10, *r* = 0.57, 95% CI [0.48; 0.66]; *I*^2^ = 92.9%); as for the subgroup of associations, ICD-11 traits model and DSM-5 AMPD traits model (*k* = 44, *r* = 0.63, 95% CI [0.57; 0.69], *I*^2^ = 97.9%). The convergent validity between measures of PD severity and traits between one diagnostic system and another has been demonstrated in this review and they can probably be used interchangeably because they also measure the same constructs. Future research can address the limitations of this study and review the evidence for the discriminant validity of these measures.

## 1 Introduction

Personality involves the way a person behaves, interprets themselves, perceives life, other people and situations; while PD is a marked alteration in personal and social functioning (1). The construct of personality and its pathology has always attracted the interest of mental health professionals because it is linked to other conditions or clinically relevant results. In the last 10 years, or even more, personality disorder has been conceived in a dimensional way in the most used diagnostic systems to improve their validity and clinical usefulness (2). In both diagnostic systems for the dimensional diagnosis of PD, two similar steps are followed: identification of the level of severity of PD dysfunction and assignment of the accompanying stylistic features (1, 3). Both steps reflect the most influential paradigms in personality psychopathology. Thus severity reflects the current state of basic internal capabilities; and trait domains, the stylistic dispositions with which severity probably interacts bidirectionally (4, 5). Supplementary Table S1 shows the conceptually equivalent constructs between the ICD-11 and DSM-5 models for personality disorder.

In the DSM-5 AMPD, the PD severity model is criterion A and is defined as a unidimensional spectrum of problems in the components of identity and self-direction for the self-dysfunction domain, and of problems in empathy and intimacy for the interpersonal dysfunction domain. In the ICD-11, the first diagnostic step is the severity of intra- and interpersonal functioning, similar to that of the other model; however, guidelines for manifestations (cognitive, emotional and behavioral) and deterioration (personal and social) are added (6). Small differences are also observed at the subcomponent level.^1^ For example, in the self-direction component of the DSM-5 AMPD, two additional subcomponents are evident compared to those already described in the ICD-11 severity model: (i) the use of constructive and prosocial internal norms of behavior and (ii) the capacity for productive self-reflection. Likewise, in the empathy component of the DSM-5 AMPD, two additional subcomponents to what is mentioned in the ICD-11 are also noted: (a) tolerance of different perspectives and (b) understanding of the effects of one’s own behavior on others. Finally, regarding intimacy, the ICD-11 severity model emphasizes the ability to manage conflicts in relationships; while in the DSM-5 AMPD there is no explicit description for it (7). On the other hand, there are differences in the terms of thresholds between the two severity models. In the ICD-11, severity ranges from: none (implicit), personality difficulty, mild PD, moderate PD, and severe PD; while PD severity in DSM-5 AMPD expands from: no impairment, some impairment, moderate impairment, severe impairment, and extreme impairment; respectively.

In the DSM-5 AMPD trait model (Criterion B) there are five trait domains: negative affectivity, detachment, disinhibition, antagonism, and psychoticism. The latter does not correspond to any trait in the ICD-11 PD trait model. The ICD-11 PD traits model includes negative affectivity, detachment, disinhibition, dissociality, and anankastia.^2^ The antagonism of the DSM-5 AMPD traits model is similar to the dissociality of the ICD-11 PD traits model; and the anankastia of this last model does not have an explicit domain in the DSM-5 AMPD traits model. Although several authors have suggested that the anankastia is the inverse of the disinhibition domain, certainly other studies have found it to be an independent domain (7, 8). Furthermore, in bipolarity it is difficult, if not impossible, to qualify the absence or very low levels of the trait. At the facet level, greater differences are evident between the two models.^3^ This may be because, for example, in the DSM-5 AMPD traits model, several facets are interstitial and/or are located in the incorrect domain (9). We mention only the facets belonging to four of the five domains because they are comparable between the models as stated above.

The negative affectivity of the DSM-5 AMPD traits model mainly includes: emotional lability, anxiety, insecurity due to separation; on the other hand, its counterpart in the ICD-11 PD traits model includes: anxiety, worry, depression, vulnerability, fear, anger, hostility, guilt, shame, intra and interpersonal pessimism, emotional lability and dysregulation, low self-esteem and self-distrust (including avoidance, dependence, envy, and worthlessness), and interpersonal mistrust. Likewise, the detachment of the DSM-5 AMPD traits model mainly includes withdrawal, avoidance of intimacy, and anhedonia; while its counterpart in the ICD-11 PD traits model includes only social detachment and emotional detachment. Similarly, the DSM-5 AMPD antagonism traits model mainly includes manipulation, deception, and grandiosity; while dissociality in the ICD-11 PD traits model includes egocentrism and lack of empathy. Finally, the disinhibition of the DSM-5 AMPD traits model mainly includes irresponsibility, impulsivity and distractibility; while its counterpart in the ICD-11 PD traits model includes impulsivity, distractibility, irresponsibility, recklessness and lack of planning.

Previous studies have described instruments to evaluate both severity and traits in both models (2, 4, 8, 10–12). These measures include the Personality Inventory for DSM-5-Brief Form-Plus (PID-5-BF+) and the Personality Inventory for DSM-5-Brief Form-Plus Modified (PID-5-BF + M), which are compatible with both trait models by integrating the psychoticism and anankastia domains. We consider these instruments only within the DSM-5 AMPD traits model because they are based on items from the Personality Inventory for DSM-5 (PID-5). Demonstrations of convergent validity —significant and substantial associations between various measures developed to measure a common construct— are a basic and minimum requirement for the validity of any psychological test (13). Several authors agree that PD severity and the trait domains of negative affectivity, detachment, dissociality/antagonism, and disinhibition in both models are conceptually equivalent (14–17); this, despite the subtle differences described in this article. As a result, measures from one model were used to report results from the other model (18–20). This is further evidence that in psychological measurement this metric is often assumed rather than directly demonstrated (13). To overcome this knowledge-practice gap, it is necessary to empirically and deeply explore the significance and the strength of association between the constructs of one model and the other.

## 2 The present review

The aim of this systematic review was to explore the convergence between measured constructs of AMPD and ICD-11 personality disorders severity and trait domains —except for the associations with anankastia, psychoticism or the borderline pattern because they are not comparable between one dimensional model and the other—. We excluded studies of convergent validity between severity and trait measures between both models because this does not have major implications in clinical practice. We also excluded associations with sub-constructs (domains/components/sub-components of severity or trait facets) because the internal structure at these sub-dimensional levels is still debated (9, 21–24). Thus, we systematically searched the literature (in any language) using four databases: Web of Science, PubMed, Scopus, and Google Scholar. Similar to a previous paper (2), we used the following keywords: ((personality) AND ((disorder*) OR (patholog*))) AND (dimension*) AND ((function*) OR (severi*)) AND ((trait*) OR (domai*)) AND ((validity) OR (assessment)) AND ((ICD) OR (International Classification of Diseases)) AND ((DSM-5) OR (Diagnostic and Statistical Manual of Mental Disorders)). For this review, the Preferred Reporting Items for Systematic reviews and Meta-Analyses [PRISMA; (25, 26)] guidelines were followed.

The search returned 5,629 results (44 from Web of Science, 30 from PubMed, 5,518 from Scopus, and 37 from Google Scholar). There were no restrictions regarding the sex, age of the participants, the type of sample used or type of informant of the measures; since we assumed that the literature collected could be austere. Only studies that presented Pearson’s correlation coefficients for the severity and trait scales of both models were included. We contacted the authors of the studies to obtain the full text of the articles when they had restricted access. The quality of evidence of the included studies was assessed using the JBI Critical Appraisal Checklist for Analytical Cross Sectional Studies (27, 28); and synthesis, with the ‘Meta’ package v. RStudio software 6.5-0-2023.09.0–463. We used only six of the eight questions in the risk of bias tool because the questions ‘Was the exposure measured in a valid and reliable manner?’ and ‘Were objective, standard criteria used to measure the condition?’ explicitly qualified etiological and risk studies.

## 3 Results

### 3.1 Description of the chosen studies

Table 1 shows the 19 studies included and covers the results on this issue in the last 6 years. In these investigations, the measures that evaluate severity from the ICD-11 PD model included: the ICD-11 Personality Disorder Severity Scale (PDS-ICD-11), its version clinician rating form (PDS-ICD-11-CRF), and the ICD-11 PD Severity Clinician Rating Form. Likewise, the instruments that measure severity from the DSM-5 AMPD model include: the Level of Personality Functioning Scale–Brief Form (LPFS-BF), its second version (LPFS-BF 2.0), its informant version (LPFS-BF 2.0-I), and the Semi-Structured Interview for Personality Functioning DSM-5 (STiP 5.1). On the other hand, the measures that examine the trait domains from the ICD-11 PD model include: four scales from the PiCD, the ICD-11 PD Traits Clinician Rating Form and the PAQ-11. Similarly, the instruments that measure the trait domains from the DSM-5 AMPD model involved: four scales of the PID-5, its short form (PID-5-SF), its brief form plus (PID- 5-BF+), its informant brief form plus (I-PID-5-BF+), and the LPFS-SR-FFM Trait Coded (LPFS-SR-FFM-TC). Supplementary Table S2 describes the scales measuring personality disorder severity and trait domains from the studies analyzed.

**Table 1:** Description of included studies.

Study	Year	Associated models of PD	ICD-11 PD measure	AMPD measure	r coefficient	Sample size	Sample type	Setting	Country	Language	Gender, % female	Age, M	Technique / informant	Quality assessment
1. Bach et al. (29)	2023	ICD-11 Severity-DSM-5 Severity	PDS-ICD-11	LPFS-BF 2.0	0.67	3,044	Community adults	Digital post	Denmark	Danish	55.8%	NR	Questionnaire / Self	Eligibility Criteria: Yes Sample and Setting: Yes Confounding Identification: Yes Confounding Management: Yes Measurement: Yes Statistics: Yes
2. Brown and Sellbom (30)	2023	ICD-11 Severity-DSM-5 Severity	PDS-ICD-11 PDS-ICD-11 PDS-ICD-11	STiP 5.1 LPFS-BF 2.0-I LPFS-BF 2.0	0.63 0.35 0.66	234	Clinical adults	Community mental health treatment	New Zealand	English	59%	NR	Interview / Clinician Questionnaire / Other Questionnaire / Self	Eligibility Criteria: Yes Sample and Setting: Yes Confounding Identification: No Confounding Management: No Measurement: Yes Statistics: Yes
3. Sellbom et al. (31)	2023	ICD-11 Severity-DSM-5 Severity	PDS-ICD-11 CRF PDS-ICD-11 CRF	LPFS-BF 2.0-I LPFS-BF 2.0	0.46 0.57	46 86	Clinical adults	Community mental health treatment	New Zealand	English	71%	36.7	Questionnaire / Clinician Questionnaire / Other Questionnaire / Self	Eligibility Criteria: Yes Sample and Setting: Yes Confounding Identification: No Confounding Management: No Measurement: Yes Statistics: Yes
4. Brown and Sellbom (32)	2022	ICD-11 Severity-DSM-5 Severity	ICD-11 PD severity ICD-11 PD severity	LPFS-BF 2.0-I LPFS-BF 2.0	0.31 0.54	311	Clinical adults	Community mental health treatment	New Zealand	English	64.30%	NR	Interview / Clinician Questionnaire / Other Questionnaire / Self	Eligibility Criteria: Yes Sample and Setting: Yes Confounding Identification: No Confounding Management: No Measurement: Yes Statistics: Yes
5. Bach et al. (33)	2021	ICD-11 Severity-DSM-5 Severity	PDS-ICD-11	LPFS-BF 2.0	0.68	515	Mixed (clinical and community) adults	Digital post	USA and New Zealand	English	50,9% community (USA) 61,5% clinical (New Zealand)	NR	Questionnaire / Self	Eligibility Criteria: Yes Sample and Setting: Yes Confounding Identification: No Confounding Management: No Measurement: Yes Statistics: Yes
6. Oltmanns and Widiger (34)	2019	ICD-11 Traits-DSM-5 Traits	PiCD_NA PiCD_DT PiCD_DL PiCD_DN	LPFS-SR-FFM-TC_NA LPFS-SR-FFM-TC_DT LPFS-SR-FFM-TC_ANT LPFS-SR-FFM-TC_DN	0.73 0.46 0.53 0.56	269	Mixed (clinical and community) adults	Digital post	USA	English	68%	31.8	Questionnaire / Self	Eligibility Criteria: Yes Sample and Setting: Yes Confounding Identification: No Confounding Management: No Measurement: Yes Statistics: Yes
7. Damovsky et al. (35)	2022	ICD-11 Traits-DSM-5 Traits	PiCD_NA PiCD_DT PiCD_DL PiCD_DN	PID-5-BF + _NA PID-5-BF + _DT PID-5-BF + _ANT PID-5-BF + _DN	0.80 0.70 0.74 0.77	939	Mixed (clinical and community) adults	Psychiatric hospital and Digital post	Germany	German	69%	32.17	Questionnaire / Self	Eligibility Criteria: Yes Sample and Setting: Yes Confounding Identification: No Confounding Management: No Measurement: Yes Statistics: Yes
8. Zimmermann et al. (36)	2022	ICD-11 Severity-DSM-5 Severity	PDS-ICD-11	LPFS-BF	0.74	1,228	Community adults	Digital post	Germany	German	50.4%	49.1	Questionnaire / Self	Eligibility Criteria: Yes Sample and Setting: Yes Confounding Identification: Yes Confounding Management: Yes Measurement: Yes Statistics: Yes
9. McCabe and Widiger (37)	2019	ICD-11 Traits-DSM-5 Traits	PiCD_NA PiCD_DT PiCD_DL PiCD_DN	PID-5_NA PID-5_DT PID-5_ANT PID-5_DN	0.86 0.78 0.81 0.89	300	Community adults	NR	USA	English	54%	36.51	Questionnaire / Self	Eligibility Criteria: Yes Sample and Setting: No Confounding Identification: No Confounding Management: No Measurement: Yes Statistics: Yes
10. Brown and Sellbom (38)	2023	ICD-11 Traits-DSM-5 Traits	ICD-11 PD Traits_ NA ICD-11 PD Traits_ DT ICD-11 PD Traits_ DL ICD-11 PD Traits_ DN ICD-11 PD Traits_ NA ICD-11 PD Traits_ DT ICD-11 PD Traits_ DL ICD-11 PD Traits_ DN	PID-5-BF + _NA PID-5-BF + _DT PID-5-BF + _ ANT PID-5-BF + _DN I-PID-5-BF + _NA I-PID-5-BF + _DT I-PID-5-BF + _ANT I-PID-5-BF + _DN	0.48 0.63 0.43 0.52 0.26 0.38 0.27 0.40	336	Clinical adults	Community mental health treatment	New Zealand	English	65.1	NR	Interview / Clinician Questionnaire / Other Questionnaire / Self	Eligibility Criteria: Yes Sample and Setting: Yes Confounding Identification: No Confounding Management: No Measurement: Yes Statistics: Yes
11. Cieciuch et al. (39)	2022	ICD-11 Traits-DSM-5 Traits	PiCD_NA PiCD_DT PiCD_DL PiCD_DN	PID-5_NA PID-5_DT PID-5_ANT PID-5_DN	0.77 0.68 0.73 0.70	597	Community adults	NR	Poland	Polish	51.4%	30.24	Questionnaire / Self	Eligibility Criteria: Yes Sample and Setting: No Confounding Identification: No Confounding Management: No Measurement: Yes Statistics: Yes
12. García et al. (40)	2022	ICD-11 Traits-DSM-5 Traits	PiCD_NA PiCD_DT PiCD_DL PiCD_DN	PID-5-SF_NA PID-5-SF_DT PID-5-SF_ANT PID-5-SF_DN	0.77 0.61 0.71 0.81	1,565	Community adults	NR	Spain	Spanish	53.8%	36.5	Questionnaire / Self	Eligibility Criteria: Yes Sample and Setting: No Confounding Identification: No Confounding Management: No Measurement: Yes Statistics: Yes
13. Sellbom et al. (41)	2022	ICD-11 Traits-DSM-5 Traits	PAQ-11_NA PAQ-11_DT PAQ-11_DL PAQ-11_DN	PID-5-BF + _NA PID-5-BF + _DT PID-5-BF + _ ANT PID-5-BF + _DN	0.72 0.58 0.48 0.57	428	Community adults	Digital post	USA	English	50.9%	45.7	Questionnaire / Self	Eligibility Criteria: Yes Sample and Setting: Yes Confounding Identification: Yes Confounding Management: Yes Measurement: Yes Statistics: Yes
14. Kim et al. (42)	2021	ICD-11 Traits-DSM-5 Traits	PAQ-11_NA PAQ-11_DT PAQ-11_DL PAQ-11_DN	PID-5-SF_NA PID-5-SF_DT PID-5-SF_ANT PID-5-SF_DN	0.74 0.67 0.70 0.57	409	Mixed (clinical and community) adults	Women’s university and Psychiatric outpatient clinic	South Korea	Korean	93.64%	23.7	Questionnaire / Self	Eligibility Criteria: Yes Sample and Setting: Yes Confounding Identification: No Confounding Management: No Measurement: Yes Statistics: Yes
15. Kerber et al. (43)	2020	ICD-11 Traits-DSM-5 Traits	PiCD_NA PiCD_DT PiCD_DL PiCD_DN	PID-5-BF + _NA PID-5-BF + _DT PID-5-BF + _ ANT PID-5-BF + _DN	0.81 0.76 0.65 0.75	493	Community adults	NR	Germany	German	70.8%	35.7	Questionnaire / Self	Eligibility Criteria: Yes Sample and Setting: No Confounding Identification: No Confounding Management: No Measurement: Yes Statistics: Yes
16. Oltmanns and Widiger (44)	2017	ICD-11 Traits-DSM-5 Traits	PiCD_NA PiCD_DT PiCD_DL PiCD_DN	PID-5_NA PID-5_DT PID-5_ ANT PID-5_DN	0.81 0.80 0.77 0.85	285	Community adults	NR	USA	English	66%	35.1	Questionnaire / Self	Eligibility Criteria: Yes Sample and Setting: No Confounding Identification: No Confounding Management: No Measurement: Yes Statistics: Yes

NR, not reported; _NA, _Negative Afectivity; _DT, _Detachment; _DL, _Dissociality; _ANT, _Antagonism; _DN, _Disinhibition; PDS-ICD-11, ICD-11 Personality Disorder Severity Scale; PDS-ICD-11-CRF, ICD-11 Personality Disorder Severity Scale Clinician Rating Form; ICD-11 PD severity, ICD-11 Personality Disorder Severity Clinician Rating Form; LPFS-BF, Level of Personality Functioning Scale– Brief Form; LPFS-BF 2.0, Level of Personality Functioning Scale– Brief Form Version 2; LPFS-BF 2.0-I, Informant’s Level of Personality Functioning Scale – Brief Form Version 2; STiP 5.1, Semi-Structured Interview for Personality Functioning DSM-5; PiCD, Personality Inventory for ICD-11; ICD-11 PD Traits, ICD-11 Personality Disorder Traits; PAQ-11, Personality Assessment Questionnaire for ICD-11; LPFS-SR-FFM-TC, LPFS-FFM Trait Coded; PID-5, Personality Inventory for DSM-5; PID-5-BF, Personality Inventory for DSM-5-Brief Form; PID-5-SF, PID-5-BF, Personality Inventory for DSM-5-Short Form; PID-5-BF+, Personality Inventory for DSM-5-Brief Form Plus; I-PID-5-BF+, Informant’s Personality Inventory for DSM-5-Brief Form Plus.

The included studies used samples from seven countries (one non-Western society) with instruments developed/adapted in six languages: Danish, English, German, Korean, Polish, and Spanish. These instruments consisted of clinician-administered interviews, and self-report and informant-report questionnaires. Four studies used clinical samples of adults (30–32, 38), eight studies used community samples of adults (29, 36, 37, 39–41, 43, 44), and four studies used mixed samples (clinical and community-based) of adults (33–35, 42). The recruitment settings were: community mental health treatment units, psychiatric hospitals, a psychiatric outpatient clinic, and a women’s college. The total sample of 16 studies involved 11,085 participants; with an average of 62.5% women, and an average age of 35.8 years. The range of the correlation coefficients r was from 0.31 to 0.74 between the severity measures of both models; and r from 0.26 to 0.89, between the trait scales of both models.

### 3.2 Quality and synthesis of studies

**Figure 1:**
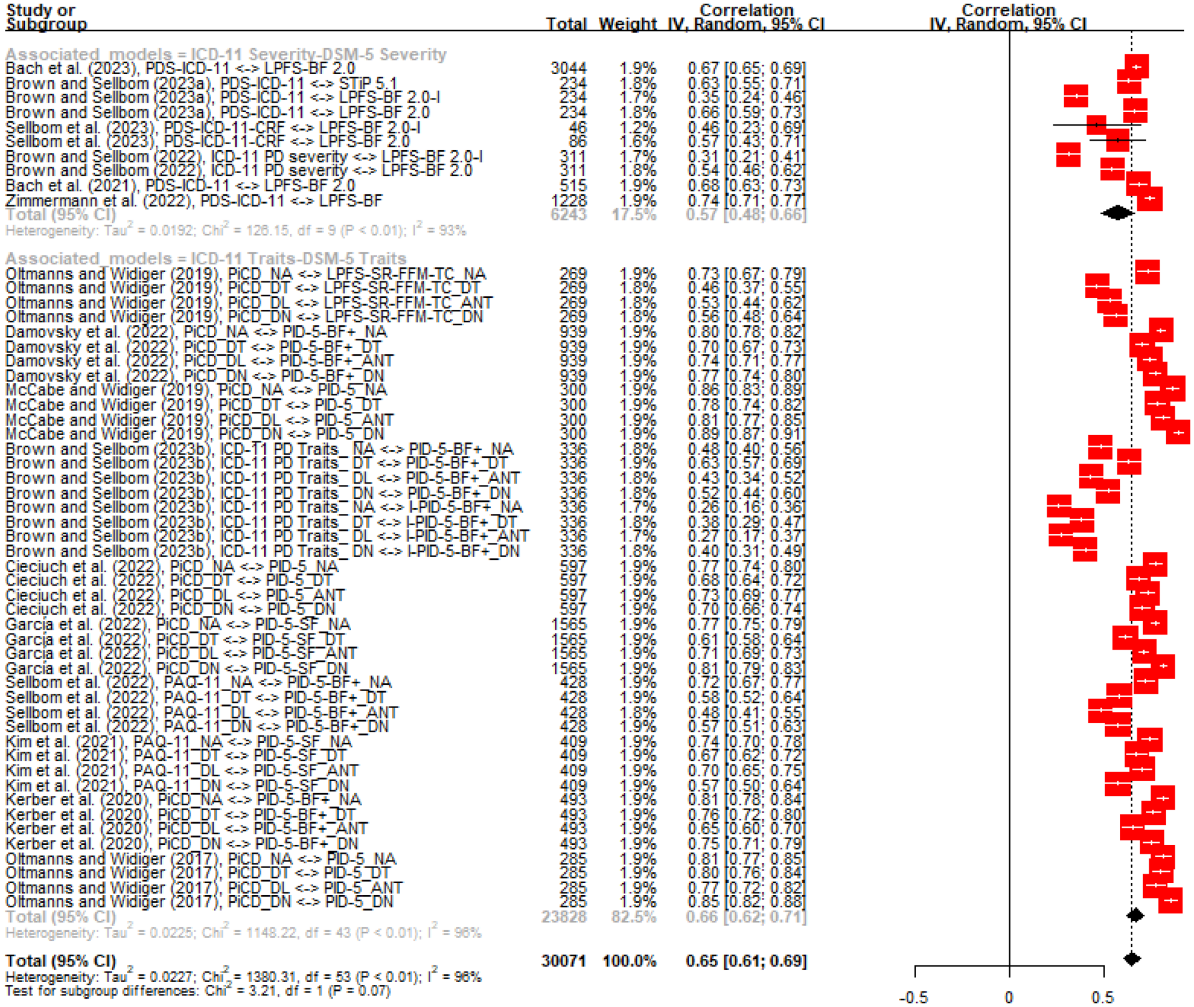
Forest plot of the reviewed studies.

Overall, the quality of the included studies was considered moderate. No studies have reported the risk of bias in more than three domains of the JBI Critical Appraisal Checklist for Analytical Cross Sectional Studies (see Supplementary Figure S1). Indeed, bias was found in 81.3% of the studies in the domains of ‘Confounding Identification’ and ‘Confounding Management’. Likewise, 25% of studies presented a risk of bias in the ‘Sample and Setting’ domain. There was no risk of bias (0%) in the domains ‘Eligibility Criteria’, ‘Measurement’, or ‘Statistics’. Four studies presented bias in ‘Sample and Setting’ (39, 40, 43, 44), as they did not adequately report demographic data, location or time period. Thirteen studies presented risk of bias in the ‘Confounding Identification’ and ‘Confounding Management’ domains (30–35, 37–40, 42–44), as baseline characteristics or prognostic factors of the results were not identified; nor were strategies such as matching or stratification used to address these confounders. Although three studies provided only one metric of interest for this study (29, 33, 36), the remainder provided two or more association coefficients that were useful for this investigation. Consequently, 54 studies in total were included in this meta-analysis. Figure 1 shows the forest plot of the studies that were meta-analyzed using the random effects method. Two subgroups are shown: the associations between the ICD-11 severity model and the DSM-5 AMPD severity model, and the associations between the ICD-11 traits model and the DSM-5 AMPD traits model.

A strong and significant degree of heterogeneity was observed in the general model (*k* = 54). That is, *τ*^2^ = 0.04, 95% CI [0.02; 0.05], which denotes a significant variance in true effects between studies (45, 46). The *I*^2^ statistic, which describes the proportion of the true variance found (46), also showed a considerable level of heterogeneity (*I*^2^ = 97.6, 95% CI [97.3%; 97.9%]). Cochrane’s *Q* also showed a significant level of heterogeneity (*χ*^2^ = 2226.80 (53), *p* = 0). The group estimator of the effect size—i.e., the summary coefficient of association—of the general model was significantly moderate (*r* = 0.62, 95% CI [0.57, 0.67], *p* < 0.0001). A significant degree of publication bias was also found using Egger’s regression test (*t* = −6.27 (52), *p* < 0.0001; see the funnel plot in Supplementary Figure S2). For the subgroup of associations between the ICD-11 severity model and DSM-5 AMPD severity model (*k* = 10), significant levels of heterogeneity were also found *τ*^2^ = 0.02, *I*^2^ = 92.9%, *χ*^2^ = 126.15. The estimated coefficient of this subgroup was significantly moderate (*r* = 0.57, 95% CI [0.48, 0.66]). In the subgroup of associations between the ICD-11 traits model and the DSM-5 AMPD traits model (*k* = 44), significant levels of heterogeneity were found *τ*^2^ = 0.04, *I*^2^ = 97.9%, *χ*^2^ = 2019.80. The estimated coefficient for this subgroup was also significantly moderate (*r* = 0.63, 95% CI [0.57, 0.69]). Finally, there was no significant difference between the associations found in these two subgroups (*χ*^2^ = 1.25 (1), *p* = 0.26). However, there is evidence that sample type and language moderated the overall effect size (*χ*^2^ = 50.7 (2), *p* < 0.0001 and *χ*^2^ = 14.27 (5), *p* = 0.01; respectively).

We also performed further analyzes of each of the trait domains as subgroups (see Supplementary Figure S3). For the subgroup of Negative Affectivity associations between the ICD-11 model and the DSM −5, a significant level of heterogeneity was found *τ*^2^ = 0.03, *I*^2^ = 95.2%, *χ*^2^ = 206.22. The coefficient estimate for this subgroup was significantly high (*r* = 0.71, 95% CI [0.61, 0.81]). Likewise, for the subgroup of Detachment associations between the ICD-11 model and the DSM −5, a significant level of heterogeneity was found *τ*^2^ = 0.04, *I*^2^ = 97.7%, *χ*^2^ = 432.20. The coefficient estimate for this subgroup was significantly moderate (*r* = 0.59, 95% CI [0.48, 0.71]). Similarly, for the subgroup of Dissociality/Antagonism associations between the ICD-11 model and the DSM-5, a significant level of heterogeneity was found *τ*^2^ = 0.06, *I*^2^ = 98.4%, *χ*^2^ = 636.14. The coefficient estimate for this subgroup was significantly moderate (*r* = 0.55, 95% CI [0.41, 0.70]). Also, for the subgroup of Disinhibition associations between the ICD-11 model and the DSM-5, a significant level of heterogeneity was found *τ*^2^ = 0.02, *I*^2^ = 97.1%, *χ*^2^ = 349.06. The coefficient estimate for this subgroup was significantly moderate (*r* = 0.68, 95% CI [0.58, 0.77]). Finally, there was no significant difference in these four subgroups (*χ*^2^ = 4.23 (3), *p* = 0.24).

## 4 Discussion

To our knowledge, this review is the first study to meta-analytically examine the convergence between the measures that evaluate PD from the new dimensional models of the two most used diagnostic standards in the world, the ICD-11 and the DSM-5 AMPD. In general, our findings indicate moderate convergence between these instruments, both for the severity and trait models. Although a high summary association would be more satisfactory —given that these instruments conceptually measure the same constructs— the results may already indicate empirical evidence for the interchangeable usefulness of these measures between one model and another. Publication bias can occur for various reasons, including heterogeneity in the methodology of studies in the meta-analysis (45), as presented here. Our results align with those described in more extensive non-meta-analytic reviews that included the convergent validity of the LPFS, and its derivatives, with other self-reported measures of PD severity (4, 24, 47). Likewise, our findings are similar to those of reviews that reported adequate levels of convergent validity between PID-5, and its derivatives, with other measures of maladaptive traits (8, 47, 48). The literature described in these reviews of the DSM-5 AMPD model instruments in relation to the ICD-11 PD model measures was extremely scarce and an update of the evidence was necessary.

The main strength of this research was the inclusion of gray literature [e.g., (31, 38)], and texts of articles in languages other than English [e.g., (35)]. However, this study has several limitations to declare. Regarding the evidence included in this review, most studies used small samples and the methodology was predominantly based on self-report questionnaires instead of using multimethod designs. Previous studies have already warned about these practices that limit the adequate interpretation of evidence (23, 49). Our study quality assessment tool is the most used by researchers because it is brief (50); however, for the same reason it may not adequately address all the shortcomings of the studies. Another limitation of the included studies was the majority use of community samples, in which the few vulnerabilities associated with PD may not reflect the exact relationship metrics that interest us. Regarding the limitations of the review processes used, we were unable to access relevant data from two studies (51, 52) because of the lack of response from the authors or the failure to understand our requirement. Likewise, we could not perform moderator analyses because the number of studies with the same measure or another possible moderator was insufficient. However, we assert that none of these methodological limitations would change the general inferences of this review. Future research could address these limitations or conduct discriminant validity analyses to complete evidence of the construct validity of the measures of one or another dimensional model of PD.

## Author contributions

LH-O: Conceptualization, Writing – original draft. TC-R: Methodology, Supervision, Writing – review & editing. JT: Conceptualization, Supervision, Validation, Writing – review & editing. DR-C: Conceptualization, Formal analysis, Methodology, Writing – original draft.

## Footnotes

^1^In the ICD-11 PD severity model there are no components or subcomponents as such, thus the comparisons with the other model described in this article are based on the guidelines provided for the identification of PD severity.

^2^An additional qualifier is also included, the borderline pattern, which is not a dimensional trait, but rather a specifier associated with a diagnostic category from the previous edition of this diagnostic system.

^3^There are no facets in the ICD-11 PD traits model, and comparisons with the other model described in this work are based on the guidelines provided for each trait domain.

## References

[1] World Health Organization. Personality disorders and related traits In: Author, editor. ICD-11 clinical descriptions and diagnostic requirements. Geneva: WHO Department of Mental Health and Substance Use (2022).

[2] Hualparuca-Olivera L Caycho-Rodríguez T. Diagnostic accuracy of severity measures of ICD-11 and DSM-5 personality disorder: clarifying the clinical landscape with the most up-to-date evidence. Front Psych. (2023) 14:1209679. doi: 10.3389/fpsyt.2023.1209679, PMID: 37324826 PMC10265646

[3] American Psychiatric Association. Diagnostic and statistical manual of mental disorders (DSM-5-TR™). Washington, DC: American Psychiatric Publishing (2022).

[4] Zimmermann J Hopwood CJ Krueger RF. The DSM-5 level of personality functioning scale In: Krueger RF Blaney PH, editors. Oxford textbook of psychopathology. Oxford, UK: Oxford University Press (2023).

[5] Sharp C Miller JD. Ten-year retrospective on the DSM-5 alternative model of personality disorder: seeing the forest for the trees. Personal Disord. (2022) 13:301–4. doi: 10.1037/per0000595, PMID: 35787110

[6] Bach B Vestergaard M. Differential diagnosis of ICD-11 personality disorder and autism Spectrum disorder in adolescents. Children. (2023) 10:992. doi: 10.3390/children10060992, PMID: 37371224 PMC10297099

[7] Bach B Mulder R. (2022) “Empirical foundation of the ICD-11 classification of personality disorders,” In: Huprich SK, editor. Personality disorders and pathology: integrating clinical assessment and practice in the DSM-5 and ICD-11 era. American Psychological Association Washington.

[8] Freilich CD Krueger RF Hobbs KA Hopwood CJ Zimmermann J. The DSM- 5 maladaptive trait model for personality disorders In: Krueger RF Blaney PH, editors. Oxford textbook of psychopathology. New York, NY: Oxford University Press (2023). 604–27.

[9] Clark LA Watson D. The trait model of the DSM-5 alternative model of personality disorder (AMPD): a structural review. Personal Disord. (2022) 13:328–36. doi: 10.1037/per0000568 35787115

[10] Oltmanns JR. Personality traits in the international classification of diseases 11th revision (ICD-11). Curr Opin Psychiatry. (2021) 34:48–53. doi: 10.1097/YCO.0000000000000656, PMID: 33252429

[11] Hualparuca-Olivera L Ramos DN Arauco PA Coz RM. Integrative dimensional personality inventory for ICD-11: development and evaluation in the Peruvian correctional setting. Liberabit. (2022) 28:e540–13. doi: 10.24265/liberabit.2022.v28n1.05

[12] Birkhölzer M Schmeck K Goth K. Assessment of criterion A. Curr Opin Psychol. (2021) 37:98–103. doi: 10.1016/j.copsyc.2020.09.009, PMID: 33099168

[13] Duckworth AL Kern ML. A meta-analysis of the convergent validity of self-control measures. J Res Pers. (2011) 45:259–68. doi: 10.1016/j.jrp.2011.02.004, PMID: 21643479 PMC3105910

[14] Mulder R. ICD-11 personality disorders: utility and implications of the new model. Front Psych. (2021) 12:709. doi: 10.3389/fpsyt.2021.655548, PMID: 34040555 PMC8141634

[15] Bach B Bernstein DP. Schema therapy conceptualization of personality functioning and traits in ICD-11 and DSM-5. Curr Opin Psychiatry. (2019) 32:38–49. doi: 10.1097/YCO.0000000000000464, PMID: 30299307

[16] Bach B First MB. Application of the ICD-11 classification of personality disorders. BMC Psychiatry. (2018) 18:1–14. doi: 10.1186/s12888-018-1908-3 30373564 PMC6206910

[17] Töre T. Comparative examination of ICD-11 and DSM-5 alternative model in personality disorders. Psikiyatr Guncel Yaklasimlar. (2023) 15:189–202. doi: 10.18863/pgy.1071669

[18] Hutsebaut J Weekers LC Tuin N Apeldoorn JSP Bulten E. Assessment of ICD-11 personality disorder severity in forensic patients using the semi-structured interview for personality functioning DSM-5 (STiP-5.1): preliminary findings. Front Psych. (2021) 12:505. doi: 10.3389/fpsyt.2021.617702, PMID: 33935824 PMC8085303

[19] Gamache D Savard C Leclerc P Payant M Berthelot N Côté A. A proposed classification of ICD-11 severity degrees of personality pathology using the self and interpersonal functioning scale. Front Psych. (2021) 12:292. doi: 10.3389/fpsyt.2021.628057, PMID: 33815167 PMC8012561

[20] Fang S Ouyang Z Zhang P He J Fan L Luo X. Personality inventory for DSM-5 in China: evaluation of DSM-5 and ICD-11 trait structure and continuity with personality disorder types. Front Psych. (2021) 12:635214. doi: 10.3389/fpsyt.2021.635214, PMID: 33841206 PMC8033014

[21] Sleep CE Lynam DR Miller JD. Personality impairment in the DSM-5 and ICD-11: current standing and limitations. Curr Opin Psychiatry. (2021) 34:39–43. doi: 10.1097/YCO.0000000000000657, PMID: 33252428

[22] Sleep CE Lynam DR. The problems with criterion a: a comment on Morey et al. (2022). Personal Disord. (2022) 13:325–7. doi: 10.1037/per0000585 35787114

[23] Bagby RM Zahid A. Revising the trait model of the alternative model of personality disorders: comment on Clark and Watson’s structural review. Personal Disord. (2022) 13:337–9. doi: 10.1037/per0000587 35787116

[24] Morey LC McCredie MN Bender DS Skodol AE. Criterion a: level of personality functioning in the alternative DSM-5 model for personality disorders. Personal Disord. (2022) 13:305–15. doi: 10.1037/per0000551, PMID: 35787111

[25] Page MJ Moher D Bossuyt PM Boutron I Hoffmann TC Mulrow CD. PRISMA 2020 explanation and elaboration: updated guidance and exemplars for reporting systematic reviews. BMJ. (2021) 372:n160. doi: 10.1136/bmj.n160, PMID: 33781993 PMC8005925

[26] Page MJ McKenzie JE Bossuyt PM Boutron I Hoffmann TC Mulrow CD. The PRISMA 2020 statement: an updated guideline for reporting systematic reviews. J Clin Epidemiol. (2021) 134:178–89. doi: 10.1016/j.jclinepi.2021.03.001, PMID: 33789819

[27] Moola S Munn Z Tufanaru C Aromataris E Sears K Sfetc R. Chapter 7: systematic reviews of etiology and risk In: Aromataris E Munn Z, editors. JBI manual for evidence synthesis. Adelaide: JBI (2020).

[28] Barker TH Stone JC Sears K Klugar M Leonardi-Bee J Tufanaru C. Revising the JBI quantitative critical appraisal tools to improve their applicability: an overview of methods and the development process. JBI Evid Synth. (2023) 21:478–93. doi: 10.11124/JBIES-22-00125, PMID: 36121230

[29] Bach B Simonsen E Kongerslev MT Bo S Hastrup LH Simonsen S. ICD-11 personality disorder features in the danish general population: cut-offs and prevalence rates for severity levels. Psychiatry Res. (2023) 328:115484. doi: 10.1016/j.psychres.2023.115484, PMID: 37748238

[30] Brown TA Sellbom M. Further validation of the personality disorder severity for ICD-11 (PDS-ICD-11) scale in a community mental health sample. Psychol Assess. (2023) 35:706–14. doi: 10.1037/pas0001253, PMID: 37384513

[31] Sellbom M Brown TA Bach B. Development and psychometric evaluation of the personality disorder severity ICD-11 (PDS-ICD-11) clinician-rating form. Personal Ment Health. (2023). doi: 10.1002/pmh.1596, PMID: 37941508

[32] Brown TA Sellbom M. Examining the reliability and validity of the ICD-11 personality disorder severity diagnosis. Aust N Z J Psychiatry. (2022) 57:1043–51. doi: 10.1177/00048674221136457 36384302

[33] Bach B Brown TA Mulder RT Newton-Howes G Simonsen E Sellbom M. Development and initial evaluation of the ICD-11 personality disorder severity scale: PDS-ICD-11. Personal Ment Health. (2021) 15:223–36. doi: 10.1002/pmh.1510, PMID: 34002530

[34] Oltmanns JR Widiger TA. Evaluating the assessment of the ICD-11 personality disorder diagnostic system. Psychol Assess. (2019) 31:674–84. doi: 10.1037/pas0000693, PMID: 30628821 PMC6488396

[35] Damovsky F Zettl M Zimmermann J Herbold W Curtius T Bücker S. The personality inventory for ICD-11 (PiCD): reliability and validity of the german version in a clinical and non-clinical sample. Psychother Psychosom Med Psychol. (2022) 73:62–9. doi: 10.1055/a-1826-1888, PMID: 36055254

[36] Zimmermann J Falk C Wendt LP Spitzer C Fischer F Bach B. Validating the German version of the personality disorder severity-ICD-11 scale using nominal response models. Psychol Assess. (2022) 35:257–68. doi: 10.1037/pas0001199, PMID: 36455031

[37] McCabe GA Widiger TA. A comprehensive comparison of the ICD-11 and DSM-5 section III personality disorder models. Psychol Assess. (2020) 32:72–84. doi: 10.1037/pas0000772, PMID: 31580095

[38] Brown TA Sellbom M. Examining the validity and factor structure of the ICD-11 trait domains In: Brown TA, editor. Elucidating the validity and utility of the ICD-11 personality disorder diagnosis: A multi-method examination. Otago: University of Otago (2023). 93–131.

[39] Cieciuch J Łakuta P Strus W Oltmanns JR Widiger T. Assessment of personality disorder in the ICD-11 diagnostic system: polish validation of the personality inventory for ICD-11. Psychiatr Pol. (2022) 56:1185–202. doi: 10.12740/PP/OnlineFirst/138563, PMID: 37098193

[40] García LF Aluja A Urieta P Gutierrez F. High convergent validity among the five-factor model, PID-5-SF, and PiCD. Personal Disord Theory Res Treat. (2022) 13:119–32. doi: 10.1037/per0000486, PMID: 35286125

[41] Sellbom M Chiasson PM Brown TA Bach B. Examining the construct validity of the personality assessment questionnaire for ICD-11 (PAQ-11) personality trait domains in a community sample. Personal Ment Health. (2022) 17:197–207. doi: 10.1002/pmh.1573, PMID: 36527327

[42] Kim Y-R Tyrer P Hwang S-T. Personality assessment questionnaire for ICD-11 personality trait domains: development and testing. Personal Ment Health. (2021) 15:58–71. doi: 10.1002/pmh.1493, PMID: 32638542

[43] Kerber A Schultze M Müller S Rühling RM Wright AGC Spitzer C. Development of a short and ICD-11 compatible measure for DSM-5 maladaptive personality traits using ant Colony optimization algorithms. Assessment. (2020) 29:467–87. doi: 10.1177/1073191120971848, PMID: 33371717 PMC8866743

[44] Oltmanns JR Widiger TA. A self-report measure for the ICD-11 dimensional trait model proposal: the personality inventory for ICD-11. Psychol Assess. (2017) 30:154–69. doi: 10.1037/pas0000459, PMID: 28230410 PMC5930359

[45] Oltmanns JR Oltmanns TF. Self–other agreement on ratings of personality disorder symptoms and traits: three meta-analyses In: Letzring TD Spain JS, editors. The Oxford handbook of accurate personality judgment. New York, NY: Oxford University Press (2021). 276–93.

[46] Higgins JPT. Commentary: heterogeneity in meta-analysis should be expected and appropriately quantified. Int J Epidemiol. (2008) 37:1158–60. doi: 10.1093/ije/dyn204, PMID: 18832388

[47] Zimmermann J Kerber A Rek K Hopwood CJ Krueger RF. A brief but comprehensive review of research on the alternative DSM-5 model for personality disorders. Curr Psychiatry Rep. (2019) 21:92. doi: 10.1007/s11920-019-1079-z, PMID: 31410586

[48] Barchi-Ferreira Ana Maria B Osório FL. The personality inventory for DSM-5: psychometric evidence of validity and reliability—updates. Harv Rev Psychiatry. (2020) 28:225–37. doi: 10.1097/HRP.0000000000000261, PMID: 32692087

[49] Natoli AP Bornstein RF. Validating the level of personality functioning scale: we don’t use multimethod research designs. PsyArXiv. (2019). doi: 10.31234/osf.io/nhrd2

[50] Ma L-L Wang Y-Y Yang Z-H Huang D Weng H Zeng X-T. Methodological quality (risk of bias) assessment tools for primary and secondary medical studies: what are they and which is better? Mil Med Res. (2020) 7:7. doi: 10.1186/s40779-020-00238-8, PMID: 32111253 PMC7049186

[51] Oltmanns JR Widiger TA. The five-factor personality inventory for ICD-11: a facet-level assessment of the ICD-11 trait model. Psychol Assess. (2020) 32:60–71. doi: 10.1037/pas0000763, PMID: 31414852 PMC6928398

[52] Somma A Gialdi G Fossati A. Reliability and construct validity of the personality inventory for ICD-11 (PiCD) in Italian adult participants. Psychol Assess. (2020) 32:29–39. doi: 10.1037/pas0000766, PMID: 31414851

