# Corrigendum: Convergence between the dimensional PD models of ICD-11 and DSM-5: a meta-analytic approach

**DOI:** 10.3389/fpsyt.2024.1366574

**Published:** 2024-03-21

**Authors:** Luis Hualparuca-Olivera, Tomás Caycho-Rodríguez, Julio Torales, Dayana Ramos-Campos

**Affiliations:** ^1^ Escuela de Psicología, Universidad Continental, Huancayo, Peru; ^2^ Facultad de Psicología, Universidad Científica del Sur, Lima, Peru; ^3^ Department of Medical Psychology, School of Medical Sciences, Universidad Nacional de Asunción, San Lorenzo, Paraguay; ^4^ Department of Research, Psychological Science, Uncanny, Huancayo, Peru

**Keywords:** ICD-11, DSM-5, personality disorder, dimensional models, severity, traits, convergent validity, meta-analysis

In the published article, there was an error in Table 1, *7. Damovsky et al. (35)*, Column 6. Two incorrect r coefficients were mentioned. Instead of “0.14” and “0.00”, they should be “0.70” and “0.74”, respectively.

In the published article, there was an error in **Figure 1**. The figure showed incorrect correlation indices for ‘Damovsky et al. (2022), PiCD_DT <-> PID-5-BF+_DT’ and for ‘Damovsky et al. (2022), PiCD_DL <-> PID-5-BF+_ANT’. Instead of “0.14” and “0.00”, it should be “0.70” and “0.74”, respectively.

The correct **Figure 1** appears below.

**Figure 1:**
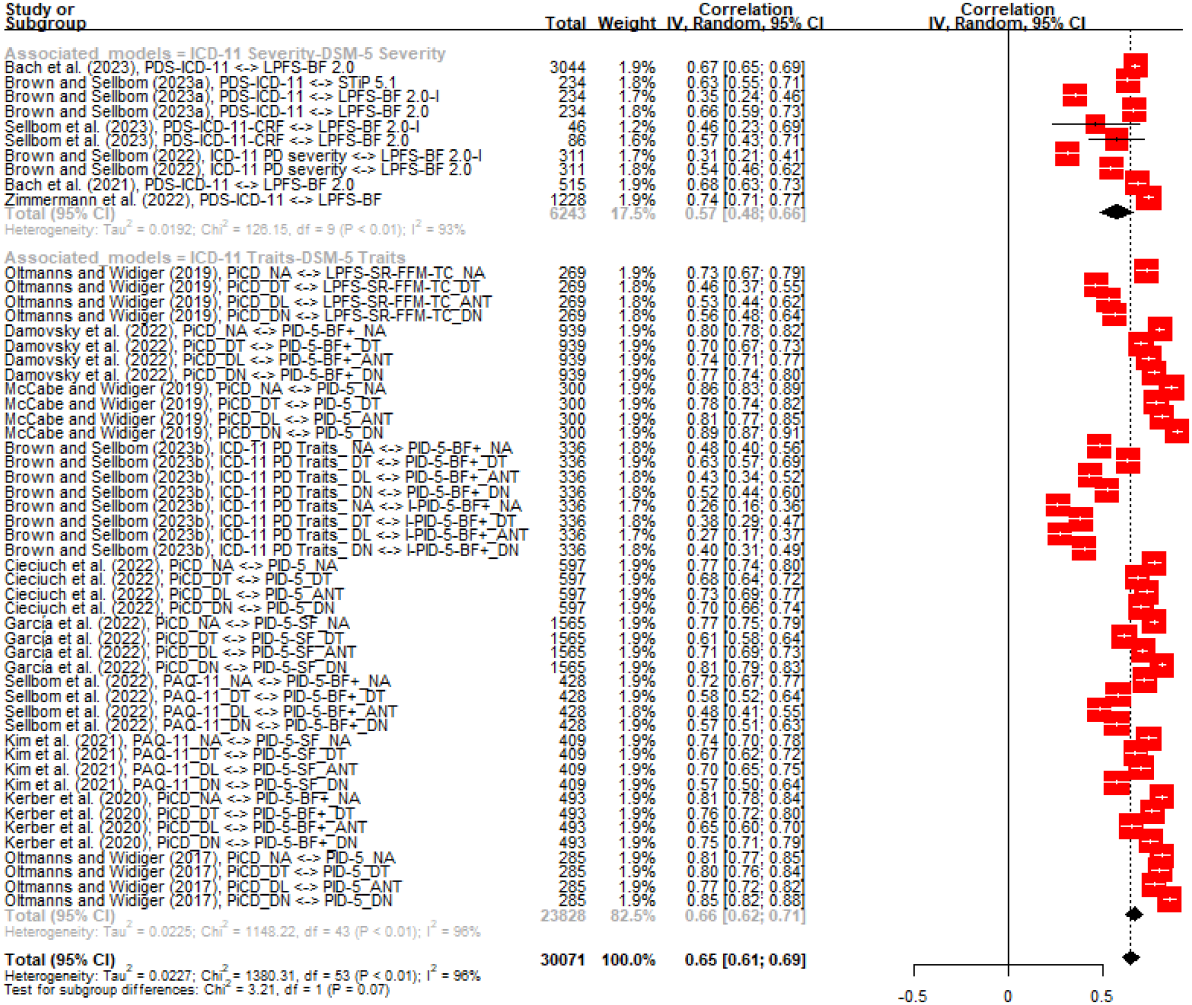
.

In the published article, there was an error in Supplementary Figure S3. The figure showed incorrect correlation indices for ‘Damovsky et al. (2022), PiCD_DT <-> PID-5-BF+_DT’ and for ‘Damovsky et al. (2022), PiCD_DL <-> PID-5-BF+_ANT’. Instead of “0.14” and “0.00”, it should be “0.70” and “0.74”, respectively.

In the published article, there was an error in **3 Results**, *3.1 Description of the chosen studies*, Paragraph 2. This sentence previously stated:

“The range of the correlation coefficients r was from 0.31 to 0.74 between the severity measures of both models; and r from 0 to 0.89, between the trait scales of both models.”

The corrected sentence appears below:

“The range of the correlation coefficients r was from 0.31 to 0.74 between the severity measures of both models; and r from 0.26 to 0.89, between the trait scales of both models.”

The authors apologize for these errors and state that this does not change the scientific conclusions of the article in any way. The original article has been updated.

