# Supplementary material for: Convergence between the dimensional PD models of ICD-11 and DSM-5: a meta-analytic approach
Source: Front Psychiatry. 2023 Nov 30;14:1325583. doi: 10.3389/fpsyt.2023.1325583 (PMC10719945; doi:10.3389/fpsyt.2023.1325583)
Supplement: Supplementary file 1 [file Data_Sheet_1.docx]

Supplementary Material

**Table S1 | ICD-11 “Cross Walk” for DSM-5 Alternative Model of Personality Disorders**

| ICD-11 severity of personality dysfunction | DSM-5 criterion A: level of personality functioning |
| --- | --- |
| None | 0) No impairment (Healthy Functioning) |
| QE50.7: Personality difficulty | 1) Some impairment |
| 6D10.0: Mild personality disorder | 2) Moderate impairment |
| 6D10.1: Moderate personality disorder | 3) Severe impairment |
| 6D10.2: Severe personality disorder | 4) Extreme impairment |
| ICD-11 trait domain qualifiers | DSM-5 criterion B: trait domains |
| 6D11.0: Negative affectivity | Negative affectivity |
| 6D11.1: Detachment | Detachment |
| 6D11.2: Dissociality | Antagonism |
| 6D11.3: Disinhibition | Disinhibition |
| 6D11.4: Anankastia | [Rigid perfectionism]^a^ |
| 6A22: [Schizotypal disorder]^b^ | Psychoticism |

^a^ This is a facet from the domain of (low) Disinhibition

^b^ This is a diagnosis from Schizophrenia or other primary psychotic disorders

**Table S2 | Measures included meta-analysis for assessment of PD severity and trait domains**

| Measure | Construct | Method | Items | Scales |
| --- | --- | --- | --- | --- |
| PDS-ICD-11 | ICD-11 severity | Self-report | 14 | 1 |
| PDS-ICD-11-CRF | ICD-11 severity | Expert rating | 14 | 1 |
| ICD-11 PD severity | ICD-11 severity | Expert rating | 1 | 1 |
| LPFS-BF | DSM-5 severity | Self-report | 12 | 2 |
| LPFS-BF 2.0 | DSM-5 severity | Self-report | 12 | 2 |
| LPFS-BF 2.0-I | DSM-5 severity | Informant report | 12 | 2 |
| STiP 5.1 | DSM-5 severity | Structured interview | 12 | 1/4 |
| PiCD | ICD-11 traits | Self-report | 60 | 5 |
| ICD-11 PD Traits | ICD-11 traits | Expert rating | 29 | 6 |
| PAQ-11 | ICD-11 traits | Self-report | 17 | 5 |
| PID-5 | DSM-5 traits | Self-report | 220 | 5/25 |
| PID-5-SF | DSM-5 traits | Self-report | 100 | 5/25 |
| PID-5-BF+ | DSM-5 traits | Self-report | 36 | 6/18 |
| I-PID-5-BF+ | DSM-5 traits | Informant report | 36 | 6/18 |
| LPFS-SR-FFM-TC | DSM-5 traits | Self-report | 80 | 5 |

*Note*: PDS-ICD-11 = ICD-11 Personality Disorder Severity Scale, PDS-ICD-11-CRF = ICD-11 Personality Disorder Severity Scale Clinician Rating Form, ICD-11 PD severity = ICD-11 Personality Disorder Severity Clinician Rating Form, LPFS-BF = Level of Personality Functioning Scale– Brief Form , LPFS-BF 2.0 = Level of Personality Functioning Scale– Brief Form Version 2, LPFS-BF 2.0-I = Informant's Level of Personality Functioning Scale – Brief Form Version 2, STiP 5.1 = Semi-Structured Interview for Personality Functioning DSM-5, PiCD = Personality Inventory for ICD-11, ICD-11 PD Traits = ICD-11 Personality Disorder Traits, PAQ-11 = Personality Assessment Questionnaire for ICD-11, LPFS-SR-FFM-TC = LPFS-FFM Trait Coded, PID-5 = Personality Inventory for DSM-5, PID-5-BF = Personality Inventory for DSM-5-Brief Form, PID-5-SF = PID-5-BF = Personality Inventory for DSM-5-Short Form, PID-5-BF+ = Personality Inventory for DSM-5-Brief Form Plus, I-PID-5-BF+ = Informant's Personality Inventory for DSM-5-Brief Form Plus.


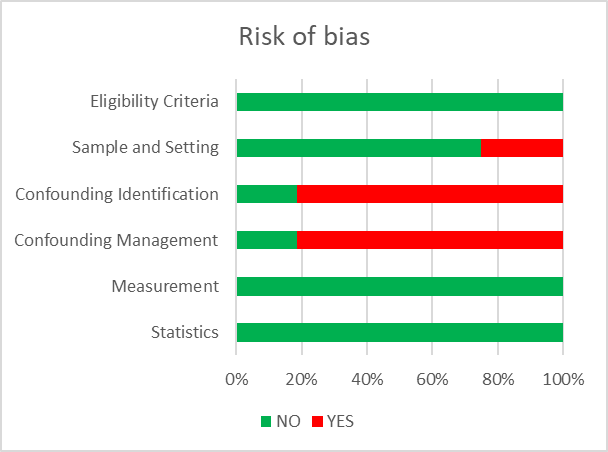


**Figure S1 | JBI Tool that Examines the Risk of Bias** **of Included Studies**


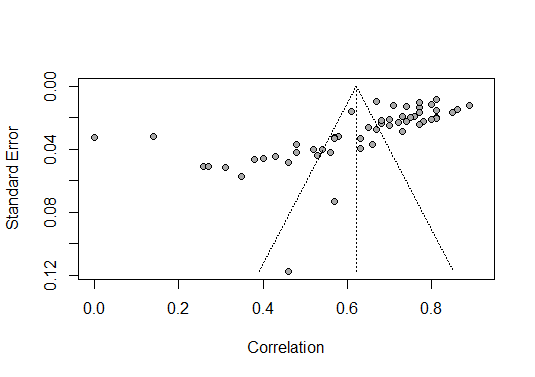


**Figure S2 | Funnel Plot for Evaluation of Publication Bias**





**Figure S3 | Forest plot of reviewed studies of associations between trait domains**
